# Dynamic Imaging of Lipid Order and Heterogeneous Microviscosity in Mitochondrial Membranes of Potato Tubers Under Abiotic Stress

**DOI:** 10.3390/membranes15100302

**Published:** 2025-10-06

**Authors:** Vadim N. Nurminsky, Svetlana I. Shamanova, Olga I. Grabelnych, Natalia V. Ozolina, Yuguang Wang, Alla I. Perfileva

**Affiliations:** 1 Siberian Institute of Plant Physiology and Biochemistry, Siberian Branch of Russian Academy of Sciences, 664033 Irkutsk, Russia; shamanova@sifibr.irk.ru (S.I.S.); grolga@sifibr.irk.ru (O.I.G.); ozol@sifibr.irk.ru (N.V.O.); alla.light@mail.ru (A.I.P.); 2 Heilongjiang Sugar Beet Engineering Technology Research Center, College of Advanced Agriculture and Ecological Environment, Heilongjiang University, Harbin 150080, China; wangyuguang@hlju.edu.cn; 3 Heilongjiang Provincial Key Laboratory of Ecological Restoration and Resource Utilization for Cold Region, College of Life Sciences, Heilongjiang University, Harbin 150080, China; 4 National Sugar Crop Improvement Centre, College of Advanced Agriculture and Ecological Environment, Heilongjiang University, Harbin 150080, China

**Keywords:** mitochondria, hyperosmotic stress, membrane microviscosity, Laurdan, generalized polarization

## Abstract

Microviscosity and lipid order are the main parameters characterizing the phase states of the membrane. Variations in microviscosity and lipid composition in a living cell may indicate serious disturbances, including various kinds of stress. In this work, the effect of hyperosmotic stress on the microviscosity of mitochondrial membranes was investigated, using potato (*Solanum tuberosum* L.) tuber mitochondria. The microviscosity of mitochondrial membranes isolated from check and stressed (9 days at 34–36 °C) tubers was estimated by determining the generalized polarization (GP) values using a Laurdan fluorescent probe in confocal microscopy studies. It was revealed that the GP distribution in mitochondria isolated from stressed tubers contained new component-characterizing membrane domains with an increased lipid order compared to the rest of the membrane. We have mapped the microviscosity of mitochondrial membranes for the first time and observed the dynamics of the membrane microviscosity of an individual mitochondrion. The hyperosmotic stress significantly influences the functional state of potato mitochondria, decreasing the substrate oxidation rate and respiratory control coefficient but increasing MitoTracker Orange fluorescence. Under hyperosmotic stress, the microviscosity of mitochondrial membranes changes, and membrane domains with increased lipid order are formed. The revealed changes open up prospects for further research on the participation of raft-like microdomains of mitochondria in plant resistance to stress factors.

## 1. Introduction

Global warming significantly impacts many biological systems. One of the effects of global warming is an increase in water consumption by agricultural crops due to increased transpiration or an increase in various types of abiotic stress, which may ultimately limit their productivity [1,2]. In this regard, abiotic stress factors are becoming primary and most problematic in modern agriculture [3].

Abiotic stress factors are capable of exerting significant effects on key plant physiological processes such as photosynthesis, biochemical pathways, and plant stress tolerance, including respiration rate. Under these types of stress, plants develop resistance by adapting various defense mechanisms. Therefore, knowledge of plant responses to water stress is becoming increasingly important as it is crucial to predict the impact of climate change on crop yields [4]. The potato is the third-largest crop in terms of human consumption after wheat and rice. It contains a lot of dietary energy, which helps to reduce hunger and improve nutrition. Unfortunately, potatoes are also very sensitive to drought stress, which affects the quality and production [5].

The plant’s response to stress is aimed at metabolic restructuring, which results in protection from stress factors. Mitochondria are the center of energy metabolism in plant cells. These are organelles involved in the process of cellular respiration, and whose main function is the production of ATP as a result of oxidative phosphorylation [6]. This process is associated with electron transport along the electron transport chain (ETC), and the transfer of protons from the mitochondrial matrix through the inner membrane into the intermembrane space, followed by the return of protons through ATP-synthase to the matrix. The function of mitochondria is closely related to their ultrastructure, which is characterized by a complex membrane architecture that determines specific submitochondrial compartments. The oxphosome architecture of the oxidative phosphorylation system (OXPHOS) in mitochondria is shown [7], which presumably contributes to an increase in the efficiency of the OXPHOS system and determines the morphology of the inner mitochondrial membrane. Mitochondrial membranes are highly dynamic and are constantly rebuilt during fusion and division of mitochondria.

Under stress, the bioenergetic functions of mitochondria weaken and become defective. In plants exposed to stress, mitochondria can be involved in the process of programmed cell death—apoptosis, as suppliers of reactive oxygen species (ROS), and a number of adaptogenic factors [8]. It is known that mitochondria play a key role in the response of plants to stress effects of various nature [9], for instance, in photorespiration, proline oxidation, and ascorbate synthesis [10]. All these phenomena are an integrated result of both increased ROS production [11] and insufficient ATP production in mitochondria [12,13]. On the other hand, under mild stress, signaling pathways involving mitochondria can be triggered that cause changes in transcription levels of nuclear genes, with subsequent cellular adaptation to stress [14].

Mitochondria are also thought to play a key role in drought adaptation of plants by acting against drought-induced oxidative stress. The mitochondrial antioxidant system, through the detoxification of superoxides and peroxides, influences redox signaling, which helps plants adapt to drought stress [15]. Therefore, according to current concepts, the function of mitochondria goes far beyond being simply the “power plant of the cell”, contributing regulatory and metabolic components in addition to energy production and dissipation throughout ontogenesis and during stress [16]. In addition, mitochondria highly participate in retrograde signaling pathways under stress conditions [17,18].

In plant cells, biomembranes are the first to respond to stress since they represent a barrier to the external environment. Membranes are known to be characterized by the properties of liquid crystals, and these can make the transition to a liquid-ordered state under physiological conditions [19]. These also exhibit heterogeneity in both composition and microviscosity [20,21].

Various types of stress impact the composition and packing of lipids in membranes. These changes help cells adapt to unfavorable conditions and maintain membrane integrity. Membrane functioning largely depends on the microviscosity of the lipid bilayer, the mobility of phospholipid molecules in the membrane, and the phase state of membrane lipids. Lipid packing in cellular membranes has a direct effect on membrane tension and microviscosity, and plays a central role in cellular adaptation, homeostasis, and disease. Membrane microviscosity, in turn, has an effect on diffusion rate, transport of molecules, activity of membrane enzymes, and synthetic processes [22]. With regard to mitochondrial membranes, microviscosity determines the activity of respiratory chain transporters, the establishment of chemiosmotic potential (ΔμH^+^) in the intermembrane space, and the activity of ATP synthase.

The heterogeneity of the cell membrane lipid composition leads to the formation of more ordered lipid domains, differing in structure and properties from the rest of the membrane [23,24,25]. At the end of the last century, the concept of dynamic, ordered micro- and nanodomains in biomembranes—so-called membrane rafts, enriched in sphingolipids and sterols as well as membrane proteins—was developed [26]. Previously, it was believed that mitochondrial membranes do not contain lipid rafts [27], although later, the presence of tightly packed microdomains in them was shown [28,29,30].

Under stress in plants, variations in lipid composition in cell-membrane rafts (microdomains) take place. Membrane rafts are known to be involved in the cell’s defense mechanisms. Therefore, the state and heterogeneity of mitochondrial membranes under various types of stress, in particular dehydration and osmotic stress, are of interest.

The aim of our investigation was to visualize the microviscosity fluctuations in mitochondrial membranes under hyperosmotic stress using mitochondria from potato tubers.

## 2. Materials and Methods

### 2.1. Plant Material and Stress Treatment

The object of this study was potato tubers (*Solanum tuberosum* L., variety Gala), stored in a refrigerator at 4 °C.

Hyperthermic and hyperosmotic stress in potato tuber cells was induced by incubating the tubers in a drying cabinet for 9 days at 34–36 °C.

### 2.2. Isolation of Mitochondria, Respiratory Measurements, and Integrity of the Outer Mitochondrial Membrane

Mitochondria from potato tubers were isolated by the generally accepted method of differential centrifugation. Self-generated Percoll density gradients (consisting of 18.5, 21, and 45% Percoll) have been used for rapid purification of crude mitochondria [31]. Due to its low diffusion constant, Percoll does not penetrate the biofilm and does not cause organelle rupture. The mitochondrial suspension was kept on ice and used to analyze the respiratory activity, the outer membrane integrity, and the physical state of mitochondrial membranes.

The respiratory activity and outer membrane integrity of purified mitochondria were determined by polarographic assay using a closed-type platinum electrode (Clark-type electrode) in a 1.4 mL polarograph cell with the aid of an Oxytherm system (Hansatech Inst., Norwich, UK) at 23–24 °C [32]. The reaction medium for determining respiratory activity contained 0.3 M sucrose, 10 mM potassium phosphate buffer (pH 7.5), 10 mM KCl, 5 mM MgCl_2_, 20 mM MOPS, 5 mM EDTA, and 0.3% BSA. The oxidation substrate was 8 mM succinate in the presence of 5 mM glutamate (to prevent oxaloacetate inhibition) and 3 μM rotenone (to inhibit electron transport through complex I of ETC). The maximal rate of substrate oxidation was measured in the presence of 100–200 μM ADP (state 3). The respiratory control coefficient (RC coefficient) was calculated by dividing *V*_3_ respiration by *V*_4_ respiration (after the conversion of ADP to ATP). The outer membrane integrity was determined by the activity of cytochrome *c* oxidase or complex IV (now reclassified as a translocase EC 7.1.1.9) by the difference in the rates of ascorbate-dependent oxygen uptake stimulated by cytochrome *c* in the absence and presence of 0.04% Triton X-100 [32]. The protein content was determined by the Lowry method [33].

### 2.3. Fluorescence Microscopy and Imaging of Mitochondrial Activity

The purity of the mitochondrial fraction was determined with the aid of visualization of mitochondrial activity by potential-dependent fluorescent dye MitoTracker Orange CMTMRos (MO, chloromethyltetramethyl rosamine) (Thermo Fisher Scientific, Waltham, MA, USA) using an inverted fluorescent microscope, AxioObserver Z1 (Carl Zeiss, Oberkochen, Germany), with a digital monochrome camera (AxioCam MRm Rev.3). The software package AxioVision Rel. 4.6 was used to capture and analyze images. Excitation/absorption was set at a maximum of 554 nm, with a fluorescence wavelength of 576 nm. MO accumulates in intact mitochondria with preserved membrane potential, and its staining intensity is potential-dependent [34].

MO was added to the mitochondrial suspension (50 μL) to a final concentration of 0.1 μM; then, the suspension was incubated for 10 min at 26 °C and analyzed. The maximum fluorescence was calculated at the points of interest minus the background (at least 100 points per variant). We also used a protonophore carbonyl cyanide m-chlorophenyl hydrazone (CCCP, 9 μM) as a negative control for MO staining in order to detect mitochondrial participation in the membrane potential (ΔΨm) generation process. Uncoupling agents such as CCCP prevent the formation of a H^+^ concentration gradient between the two sides of the inner mitochondrial membrane and cause its depolarization. This negative control is often used to confirm the specificity of fluorescent dyes when studying the electrochemical potential on the inner mitochondrial membrane [35].

### 2.4. Confocal Microscopy and the Assessment of the Physical State of Mitochondrial Membranes

Confocal microscopy and the fluorescent probe 2-dimethylamino-6-lauroylnaphthalene (Laurdan) are often used to detect changes in phospholipid ordering [36]. The amphiphilic probe Laurdan was shown to be incorporated into the membrane in the hydrophilic–hydrophobic region of the lipid bilayer [37]. Laurdan is a polarity-sensitive probe whose emission spectra change with the polarity of the environment. Depending on the lipid environment, the values of the generalized polarization (GP) of Laurdan fluorescence vary, which makes it possible to assess the degree of lipid ordering [38]. GP values can range from -1 (the most liquid phase of the membrane) to +1 (the most ordered phase). The Laurdan concentration must be low enough to avoid any effect on the acyl chains of membrane lipids and, moreover, to prevent rapid fluorescence quenching [39].

**Figure 1:**
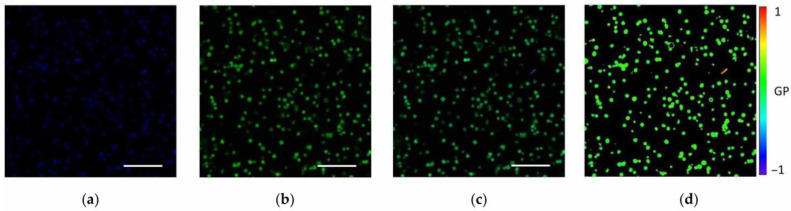
Images of isolated mitochondria fraction obtained using a confocal microscope and Laurdan probe (10 μM), *I*_ex_ = 405 nm: (**a**) channel 1 (420–460 nm); (**b**) channel 2 (470–530 nm); (**c**) both channels together (merged channels); (**d**) the corresponding GP image. Scale bar is 10 μm.

In our investigation, the physical state of mitochondrial membranes was assessed using a confocal fluorescence scanning laser microscope LSM-710 (Zeiss GmbH, Oberkochen, Germany) with a fluorescent probe Laurdan (Sigma-Aldrich, Saint Louis, MO, USA). The stock solution of Laurdan (1 mM) dissolved in DMSO (dimethyl sulfoxide) was added to the mitochondrial suspension to a final concentration of 10 μM. The suspension was incubated at 20 ± 2 °C for 10 min and analyzed on a confocal microscope. Mitochondrial fraction images (1024 × 1024 pixels) were recorded in two channels at wavelength ranges of 420–460 and 470–530 nm, respectively, *I*_ex_ = 405 nm (Figure 1). The GP values of Laurdan fluorescence for each pixel of the obtained image were computed using the image processing program ImageJ ver. 1.45s (NIH, Bethesda, MD, USA) and a special software module (macro) Generalized_Polarization_Analysis.class [40]. The GP values were adjusted with the G-factor to match the reference value measured on a spectrophotometer RF-5301PC (Shimadzu Corp., Kioto, Japan):

(1) $$GP=\frac{I_{\left(420-460\right)}-{G*I}_{\left(470-530\right)}}{I_{\left(420-460\right)}+G*I_{\left(470-530\right)}}$$

where *I*_(420–460)_ and *I*_(470–530)_ are the fluorescence intensity of Laurdan in the wavelength ranges from 420 to 460 nm and from 470 to 530 nm, respectively; *G* is the G-factor.

The G-factor can be calculated as follows:

(2) $$G=\frac{I_{et(420-460)}\times (1-{GP}_{c})}{I_{et(470-530)}\times (1+{GP}_{c})},$$

where *I_et_*_(420–460)_ and *I_et_*_(470–530)_ are the fluorescence intensities of Laurdan in the wavelength ranges from 420 to 460 nm and from 470 to 530 nm for the reference solution, which is a homogeneous solution of Laurdan (0.16 mM) in DMSO, measured on a microscope, respectively; and *GPc* is the GP value of the same reference solution, calculated using Formula (3):

(3) $${GP}_{C}=\frac{I_{sp\_et(420-460)}-I_{sp\_et(470-530)}}{I_{sp\_et(420-460)}+I_{sp\_et(470-530)}},$$

where *I_sp_et_*_(420–460)_ and *I_sp_et_*_(470–530)_ are the fluorescence intensities of Laurdan in the wavelength ranges from 420 to 460 nm and from 470 to 530 nm for a 0.16 mM Laurdan solution in DMSO, measured on the spectrophotometer, respectively. In the spectrophotometer, the samples were excited at a wavelength of 405 nm, and the emission spectra were recorded in the range from 400 to 600 nm. In our case, *GPc* = –0.29 and *G* = 0.282. Laurdan GP images of isolated mitochondria were generated based on the data obtained (Figure 1d).

**Figure 2:**
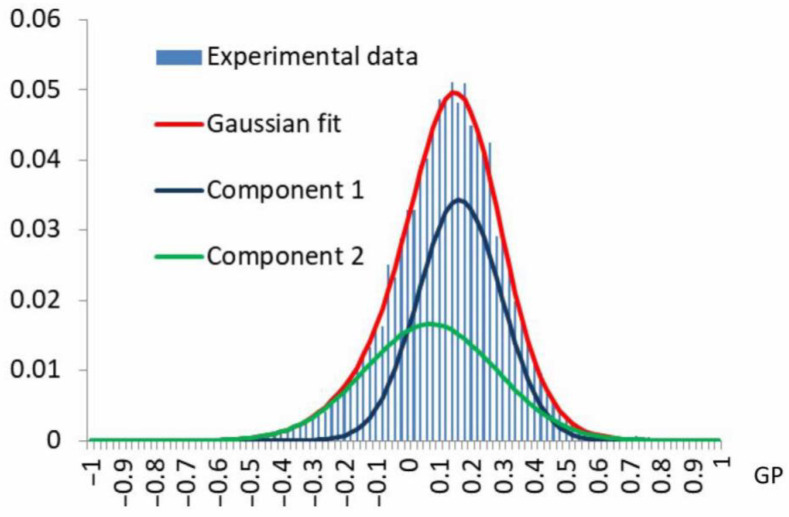
The distribution of Laurdan GP values (blue columns) calculated from the data taken from the images of the mitochondrial fraction (shown in Figure 1) and the result of deconvolution of this distribution with Gaussian fit (red line), which allows us to isolate 2 mode components (blue and green lines).

After statistical processing of the data, the distributions of GP values were presented as histograms (Figure 2).

For each histogram, a theoretical multimodal distribution was constructed as a superposition (overlay) of several Gaussian (normal) distributions, defined by the following formula:

(4) $$P\left(GP\right)=\sum_{i=1}^{k_{m}}\frac{h_{i}}{\delta_{i}}e^{-\frac{{\left(GP-m_{i}\right)}^{2}}{2{\delta_{i}}^{2}}},$$

where GP is the GP value; $k_{m}$ is the number of normally distributed modes in the multimodal distribution; $h_{i}$ is the proportion of GP values from their total number distributed by mode i in the multimodal distribution; $\delta_{i}$ is the variance for mode i; $m_{i}$ is the mathematical expectation for mode i. The values of the parameters $h_{i}$, $\delta_{i}$, $m_{i}$ for the selected values of k were determined by the least squares method. For each value of k, the value of the covariance coefficient *R*^2^ and two indicators of informative significance were calculated—the Akaike information criterion (AIC) and the Bayesian information criterion (BIC)—system indicators helping limit the number of asymptotic normal distributions. AIC was calculated using the following formula:

(5) $$AIC=\frac{2k_{p}}{n}+ln{\hat{\delta }}^{2},$$

where $k_{p}$ is the number of parameters used (estimated), *n* is the sample size, ${\hat{\delta }}^{2}$ is a consistent estimate (maximum likelihood method) of the variance in the random error of the model, equal to the ratio of the sum of the squares of the residuals to the sample size; and BIC is given by the following formula:

(6) $$BIC=n\cdot ln\left(\frac{SSE}{n}\right)+k_{p}\cdot ln(n),$$

where *SSE* is the sum of squares of the residuals. The multimodal distribution with such a value of $k_{m}$ for which *R*^2^ > 0.99 at minimum values of AIC and BIC was selected as the optimal one. Thus, a limitation on the number of mode components for describing the GP distribution was introduced.

### 2.5. Statistical Analysis

Microsoft Excel 2007 and SigmaPlot 12.5 programs were used for analysis and statistical processing of the results. The reliability of differences in fluorescence intensity between samples with check potato tubers (control) and tubers subjected to stress was estimated using the Mann–Whitney criterion, at *p* ≤ 0.001, and reliability of differences in mitochondrial activity parameters (*V*_3_, *V*_4_, RC coefficient) between samples with check potato tubers (control) and tubers subjected to stress was estimated using Tukey’s test at *p* ≤ 0.05.

## 3. Results

To induce hyperthermia and hyperosmotic stress, potato tubers were dried for 9 days at 34–36 °C. When exposed to such combined dehydrating stress conditions, the weight of potato tubers lowered by 5%, and the osmolality of cell sap elevated by 23%.

**Figure 3:**
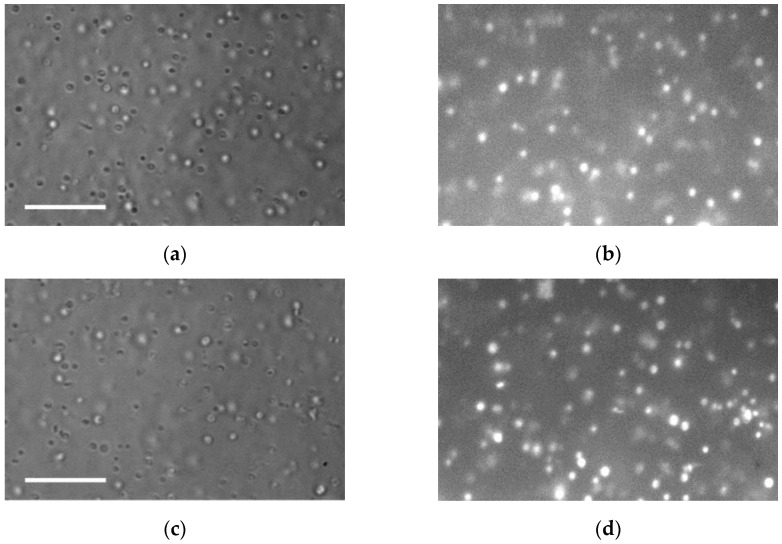
Fractions of isolated mitochondria isolated from check (**a**,**b**) and stressed (**c**,**d**) potato tubers (*S. tuberosum* L.). Light microscopy (**a**,**c**); fluorescence microscopy, MO 0.1 μM (**b**,**d**). Scale bars—10 μm.

**Figure 4:**
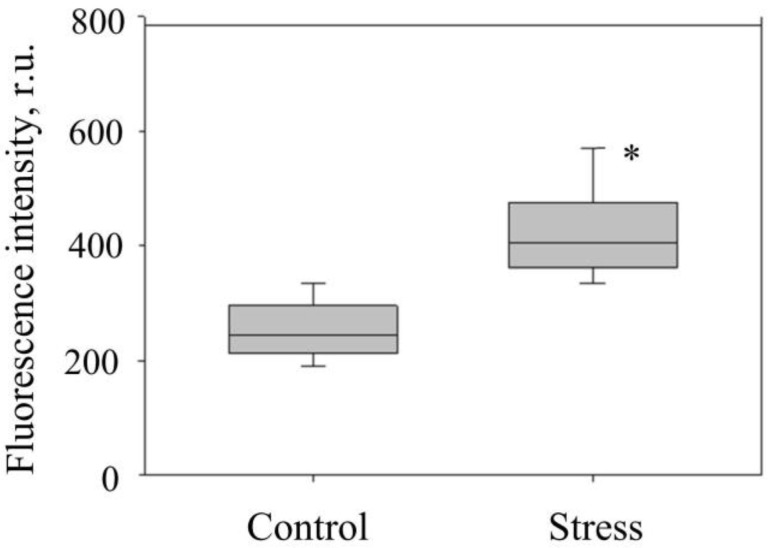
Fluorescence intensity of MO (0.1 μM) in mitochondria of potato tubers (*S. tuberosum* L.) after exposure to hyperosmotic stress. Note: r.u.—relative units; the median (Q_50_) and interquartile range [Q_25_; Q_75_] are presented, n = 3; *—statistically significant differences at *p* ≤ 0.001 (Mann–Whitney U test).

The purity of fractions of isolated mitochondria from check and stressed potato tubers was assessed using the MitoTracker probe MO (Figure 3). MO is used as a vital dye to determine the localization and activity of mitochondria in cells and to analyze isolated mitochondria using fluorescence microscopy. Under stress conditions, the fluorescence intensity of MO increased by 65% (Figure 4) (which, in our opinion, indicates an increase in ΔΨm of mitochondria under hyperosmotic stress). In order to confirm the specificity of MO binding with isolated potato mitochondria, we used protonophore CCCP that decreases ΔΨm, causing uncoupling of oxidative phosphorylation, and reduces MO fluorescence intensity. In our experiments, CCCP reduced MO fluorescence intensity in mitochondria isolated from check and stressed potato tubers by 68% and 70%, respectively.

To assess the functional activity of isolated mitochondria, the integrity of the outer membrane and the oxidative and phosphorylating activities of mitochondria were measured. The integrity of the mitochondrial outer membrane was assessed by its permeability to exogenous cytochrome *c* in the absence and presence of the detergent Triton X-100. As can be seen from Table 1, the values of intactness of mitochondria isolated from check and stressed tubers were quite high and did not differ from each other substantially. In order to determine the oxidative activity of mitochondria, succinate was used as a substrate (electron transport begins with complex II of the ETC—succinate dehydrogenase and then, via ubiquinone, electrons are transferred to complexes III and IV). It was found that under stress, the rate of phosphorylating respiration (*V*_3_) and RC coefficient decreased: *V*_3_ inhibition by 56%, and RC coefficient by 51% (Table 1).

**Figure 5:**
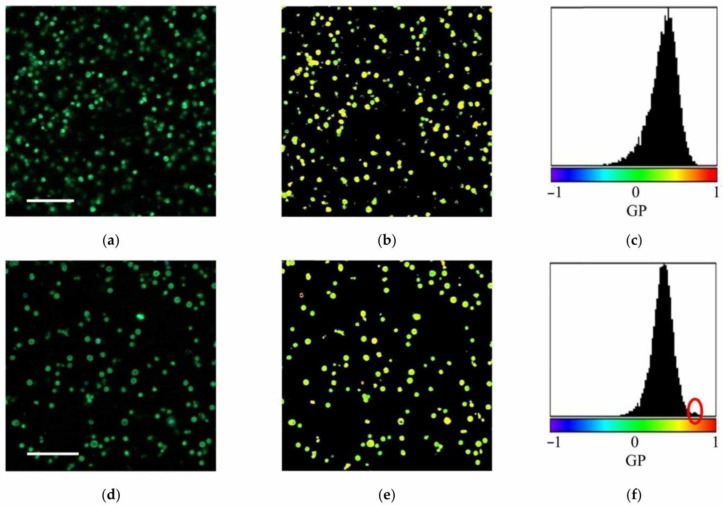
Fractions of potato mitochondria (confocal microscopy, Laurdan 10 μM) isolated from check tubers (**a**) and tubers subjected to stress (**d**); their corresponding GP images ((**b**) and (**e**), respectively) and GP value distribution histograms ((**c**) and (**f**), respectively). Scale bars are 10 μm. A red oval marks the region of the histogram responsible for the component of the GP value distribution, which characterizes membrane areas with increased microviscosity.

To analyze the microviscosity of mitochondrial membranes, individual images (Figure 5a,d) and video (sequences of images) of the fractions of isolated mitochondria from check and stressed tubers were captured. The corresponding Laurdan GP images (Figure 5b,e) and histograms of the distribution of GP values (Figure 5c,f) were constructed, and the parameters of the components of these distributions were computed.

**Figure 6:**
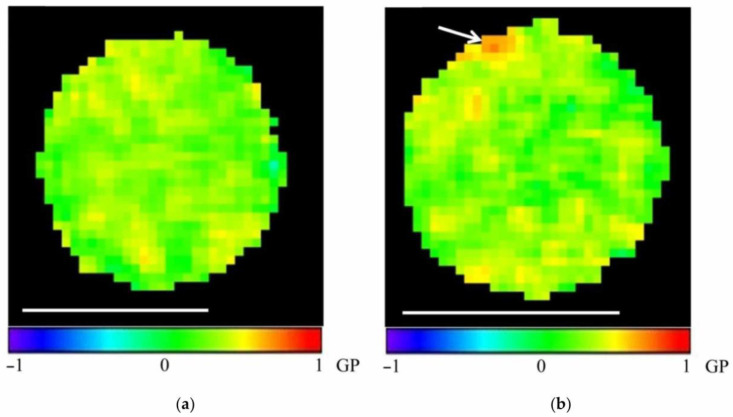
GP images of individual potato mitochondria isolated from check tubers (**a**) and tubers subjected to stress (**b**). Scale bars are 1 μm. The arrow indicates the membrane region with increased microviscosity.

**Figure 7:**
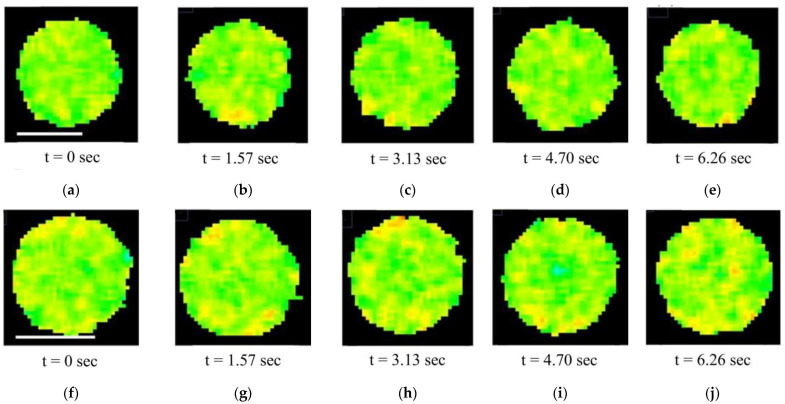
Changes in the microviscosity of the mitochondrial membranes of single organelles over time. GP images of individual potato mitochondria: check (**a**–**e**) and stressed (**f**–**j**) tubers. Scale bars—1 μm.

GP images of individual, isolated mitochondria (Figure 6) were analyzed in dynamics. In the video sequence of images of the fraction of isolated mitochondria (Video S1), it was noticed that some mitochondria were in Brownian motion, and some (stuck to the camera glass) were not moving. We analyzed mitochondria that did not change their position over 10–15 s (several frames). Therefore, GP imaging of a single, isolated mitochondrion constructed from successive images of the video sequence made it possible to visualize the membrane microviscosity of an individual organelle in dynamics (Figure 7). In addition, short video files, visually demonstrating microviscosity fluctuation in mitochondrial membranes over time, were assembled from 10 consecutive GP images of a single mitochondrion from fractions isolated from check and stressed tubers (Video S2 and Video S3, respectively).

**Figure 8:**
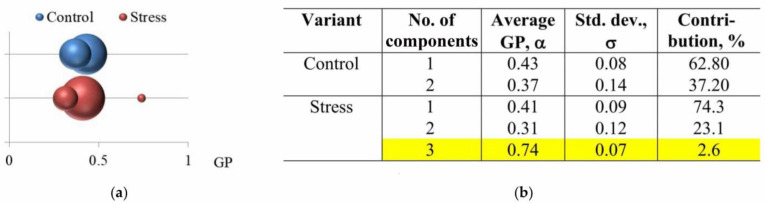
The calculated component parameters of the GP value distributions of potato mitochondrial fractions isolated from check tubers (control) and tubers subjected to hyperosmotic stress (stress): (**a**) in the form of a bubble chart (the size of the bubbe reflects the contribution of each component) and (**b**) in the form of a table (the component characterizing the more densely packed membrane regions is marked in yellow).

At the next stage of this research, the parameters of the components of the GP value distributions of mitochondria isolated from check and stressed potato tubers were computed (Figure 8). The analysis showed that the GP value distribution of mitochondria from check potato tubers contains only two components, and the distribution of mitochondria from stressed potato tubers—three components (Figure 8b). An additional minor component, characterizing more densely packed membrane regions, had the following parameters: α—0.74, and the contribution to the overall distribution was 2.6%.

## 4. Discussion

The functioning of mitochondria is largely determined by the state of their membranes, as well as by the state of the intermembrane space and matrix. Although the bioenergetics of plant cells strongly depends on the various kinds of abiotic stress, mitochondrial metabolism and the functional state of mitochondrial membranes under stress conditions are still insufficiently investigated. Mitochondria have been shown to play a central role in plant adaptation to abiotic stress that causes oxidative stress at the cellular level [41]. Mitochondria are known to regulate redox homeostasis, maintain calcium levels and energy (ATP) production through oxidative phosphorylation, and control cell death through apoptosis [42]. Studies conducted on isolated mitochondria have shown that the OXPHOS system in plants, with different life strategies, has significant similarities and species-specific organizational peculiarities [43]. It was previously found that durum wheat mitochondria have three energy dissipation systems: the plant mitochondrial ATP-sensitive potassium channel (PmitoK_ATP_); the plant uncoupling protein (PUCP); and the alternative oxidase (AOX) [10]. It was determined that these systems are capable of reducing ROS generation by mitochondria. It should be noted that potato plant is much more sensitive to drought than wheat. This is due to the stage of plant growth: potato suffers more from drought in the early period of growth and tuber formation.

The state of mitochondrial membranes is highly critical for the functioning of mitochondria under stress. Our results have shown that the mitochondria maintained their intactness even under dehydration stress (Table 1). Moreover, under influence, the mitochondrial ΔΨm increased (Figure 3 and Figure 4), which may indicate some elevation of their functional activity, mainly the functioning of their respiratory chain. However, the analysis showed that, under stress, the rate of phosphorylating respiration and the RC coefficient lowered (Table 1), which implied the uncoupling of oxidation and phosphorylation processes in the mitochondrial respiratory chain. Hyperpolarization of the inner mitochondrial membrane often accompanies the reaction of plant cells to various stress factors, including high-temperature stress [35].

The changes taking place under stress cannot help but affect the functional state of mitochondrial membranes. The inner mitochondrial membrane, containing components of the ETC, is the site where respiration directly occurs. Mitochondrial membrane microviscosity has a potential effect on the flow of electrons in the ETC and cell physiology as a whole by changing the mobility of lipid molecules in the membrane. This is due to the fact that membrane microviscosity determines the speed of molecule movement, which affects the functioning of membrane proteins/enzymes responsible for electron transport and ATP synthesis.

Various fluorescent probes are often used to assess changes in lipid ordering. Attempts to visualize membrane microviscosity using the Laurdan probe in model membranes [36] and in living cells [39,44,45,46] have been made for quite some time. Recently, Laurdan has been increasingly used to visualize microviscosity in bacterial [47], plant and animal cell membranes [48,49,50,51]. However, some articles inform that the results obtained using the Laurdan probe should be interpreted with caution, for example, in [52]. We have also used Laurdan to visualize fluctuations in the microviscosity of mitochondrial membranes from potato tubers under hyperosmotic stress.

In order to test the effect of hyperosmotic stress on the functional state of the mitochondrial membranes, we analyzed the microviscosity parameter under normal conditions and under hyperosmotic stress. Analysis of the Laurdan GP images of single, isolated mitochondria showed that their membrane is heterogeneous in terms of lipid microviscosity, containing regions with a denser packing of lipids and increased microviscosity compared to the rest of the membrane (in a ratio of approximately 2:1) (Figure 6). In addition, this heterogeneity was dynamic as there were some fluctuations in microviscosity in the mitochondrial membranes over a period of several seconds (Figure 7).

Laurdan has been used earlier to investigate the membrane dynamics of lipid vesicles under tension induced by another kind of abiotic stress, i.e., hypoosmotic effects [53]. In particular, some lowering of Laurdan GP was observed depending on osmotic stress [53]. These observations were interpreted as evidence of increased membrane tension and stretching, allowing water molecules to penetrate the hydrophobic interior of the membrane. Similar studies in living cells have shown that cells can easily withstand osmotic stress at very high dilutions of medium without evidence of membrane stretching [54].

Viscosity mapping in cells has been previously conducted with the use of fluorescent probes based on molecular rotors [22]. These probes exhibited weak fluorescence emission in low-viscosity environments, where energy is released by intramolecular rotation, but viscosity-activated “switch-on” fluorescence in high-viscosity environments, where intramolecular rotation is limited and energy is released by fluorescence emission. Using molecular rotors, it was found that the fluidity of the inner mitochondrial membrane positively correlates with respiration in a single cell [55].

In our experiments, in the mitochondrial GP value distribution of stressed potato tubers, we found a minor component characterizing more densely packed membrane regions (α: 0.74; contribution: 2.6%) (Figure 8b). In the check variant, no such components were revealed.

The molecule of lipophilic probe Laurdan consists of a lauric acid chain linked to a naphthalene moiety. The hydrophobic tail of the fatty acid inserts the probe molecule into the membrane lipid bilayer, while the naphthalene portion of the molecule localizes at the glycerol backbone of membrane phospholipids. Water molecules in lipid bilayers are tightly bound to lipid carbonyls so the mobility of lipids, along with their bound water, can influence Laurdan fluorescence properties. Laurdan structure makes it sensitive to the presence and mobility of water molecules in the lipid bilayer [49].

The interaction of the Laurdan probe with water molecules within the lipid bilayer is being intensively investigated, for example, in [56]. An approach has even been developed to analyze molecular hydration states in lipid bilayers using Laurdan fluorescence [57]. It is hypothesized that Laurdan may interact with proteins, although there is no definitive evidence of its interaction with membrane proteins [58]. Therefore, we believe that the peak of high GP values revealed (Figure 5f) is due to changes in membrane microviscosity associated primarily with lipid changes rather than protein ones. Nevertheless, it cannot be ruled out that changes in the protein composition of the membrane during stress may indirectly influence the physical state of the lipid bilayer (and regulate its microviscosity).

**Figure 9:**
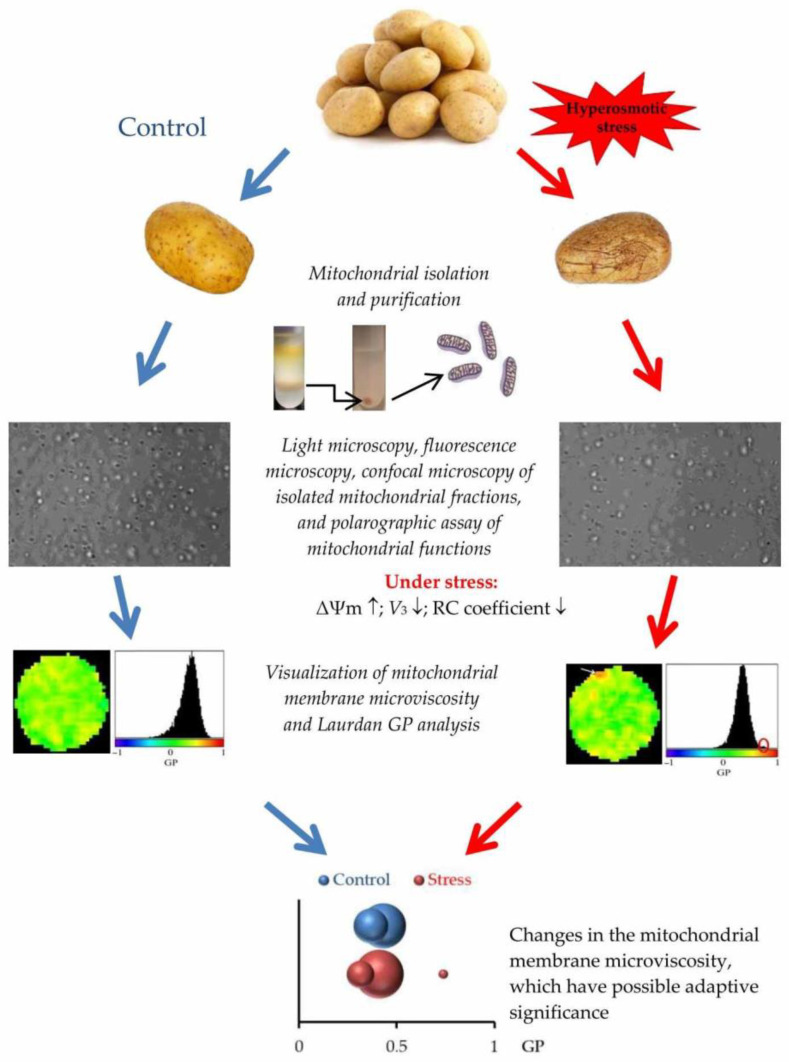
The generalized scheme reflecting the stages of the investigation conducted, which is bound up with the influence of hyperosmotic stress upon mitochondrial activity and microviscosity of mitochondrial membranes isolated from potato tubers. ΔΨm—membrane potential; GP—generalized polarization; RC—respiratory control; *V*_3_—phosphorylating respiration.

In our opinion, the identified changes in the microviscosity of the lipid bilayer may be due to increased formation of raft structures (microdomains) in the mitochondrial membranes under stress of osmotic dehydration. The microdomains are enriched in cholesterol, sphingolipids, and proteins, which are characterized by a higher packing density, resistance to detergents, and specific composition of lipids and proteins compared to other membrane regions; these have been previously found in mitochondrial membranes [28,29]. Understanding that lipid rafts can participate in plant response to stress, it can be assumed that the change in mitochondrial membrane microviscosity under hyperosmotic stress we observed is adaptive in nature. The generalized scheme of the influence of hyperosmotic stress on mitochondrial activity and microviscosity of mitochondrial membranes is shown in Figure 9.

## 5. Conclusions

Summarizing, in this work, we recorded fluctuations in mitochondrial membrane microviscosity. It was found that membrane microviscosity of a single organelle changes slightly but sharply within about 1–2 s. In our opinion, in order to obtain smoother changes in microviscosity revealed with Laurdan GP images, it is necessary to record confocal images during video recording at a higher frequency in the future.

The results obtained show that the effect of hyperthermia together with hyperosmotic stress (osmotic dehydration) significantly influences the functional state of plant mitochondria. It was found that this type of stress inhibits the respiration process, causing the uncoupling of oxidation and phosphorylation in mitochondria. Such stress has an effect on the activity of respiratory chain enzymes both directly and indirectly, through the lipid environment, changing mitochondrial membrane microviscosity.

Thus, we have shown that under hyperosmotic stress, mitochondrial membrane microviscosity changes, and densely packed microdomains (membrane rafts) presumably form in the membranes. The changes revealed open up prospects for further research on the participation of mitochondrial rafts in plant resistance to stress factors.

## Figures and Tables

**Table 1:** Functional activity and outer membrane integrity of mitochondria isolated from check tubers (control) and tubers subjected to hyperosmotic stress (stress). The oxidation substrate was succinate 8 mM. The mean values and standard deviations are presented, n = 3. *—statistically significant differences at *p* ≤ 0.05 (Tukey’s test). RC—respiratory control; *V*_3_—phosphorylating respiration; *V*_4_—non-phosphorylating respiration.

Measurable Parameter	Control	Stress
*V*_3_, nmol O_2_ × (min × mg protein)^−1^	141.0 ± 25.1	61.6 ± 12.9 *
*V*_4_, nmol O_2_ × (min × mg protein)^−1^	55.7 ± 11.3	49.5 ± 12.7
RC coefficient	2.55 ± 0.07	1.26 ± 0.06 *
Integrity, %	90 ± 2	88 ± 6

## Data Availability

The data presented in this study are available on request from the corresponding author.

## Supplementary Materials

The following supporting information can be downloaded at https://www.mdpi.com/article/10.3390/membranes15100302/s1, Video S1: video sequence of images of the mitochondrial fraction isolated from check tubers. Confocal microscopy; Laurdan probe (10 μM); *I*_ex_ = 405 nm; channel 1: 420–460 nm; channel 2: 470–530 nm; Video S2 and Video S3: membrane microviscosity fluctuations in a single mitochondrion in the fraction isolated from check and stressed potato tubers, respectively.

## Abbreviations

The following abbreviations are used in this manuscript:

AIC	Akaike information criterion
BIC	Bayesian information criterion
CCCP	Carbonyl cyanide m-chlorophenyl hydrazone
ETC	Electron transport chain
GP	Generalized polarization
Laurdan	2-dimethylamino-6-lauroylnaphthalene
MO	MitoTracker Orange CMTMRos
OXPHOS	Oxidative phosphorylation system
RC	Respiratory control
ROS	Reactive oxygen species
*V*_3_	Phosphorylating respiration
*V*_4_	Non-phosphorylating respiration
ΔμH^+^	Chemiosmotic potential
ΔΨm	Membrane potential

## References

[1] Peng S.B., Huang J.L., Sheehy J.E., Laza R.C., Visperas R.M., Zhong X.H., Centeno G.S., Khush G.S., Cassman K.G. (2004). Rice yields decline with higher night temperature from global warming. Proc. Natl. Acad. Sci. USA.

[2] Koshkin E.I., Andreeva I.V., Guseinov G.G. (2019). Impact of global climate change on productivity and stress tolerance of field crops. Agrochem.

[3] Alotaibi M. (2023). Climate change, its impact on crop production, challenges, and possible solutions. Not. Bot. Horti Agrobot. Cluj-Napoca.

[4] Atkin O., Macherel D. (2009). The crucial role of plant mitochondria in orchestrating drought tolerance. Ann. Bot..

[5] Lateef M., Naawe E.K., Hasan Z., Çalışkan M.E., Chaudhry U.K., Öztürk Z.N., Gökçe A.F. (2025). An overview of the impact of drought stress on potatoes in the era of climate change. Drought Stress.

[6] Logan D.C. (2006). The mitochondrial compartment. J. Exp. Bot..

[7] Ukolova I.V., Kondakova M.A., Kondratov I.G., Sidorov A.V., Borovskii G.B., Voinikov V.K. (2020). New insights into the organisation of the oxidative phosphorylation system in the example of pea shoot mitochondria. Biochim. Biophys. Acta—Bioenerg..

[8] Skulachev V.P. (2006). Bioenergetic aspects of apoptosis, necrosis and mitoptosis. Apoptosis.

[9] Grabelnych O.I., Borovik O.A., Tauson E.L., Pobezhimova T.P., Katyshev A.I., Pavlovskaya N.S., Koroleva N.A., Lyubushkina I.V., Bashmakov V.Y., Popov V.N. (2014). Mitochondrial energy-dissipating systems (alternative oxidase, uncoupling proteins, and external NADH dehydrogenase) are involved in development of frost-resistance of winter wheat seedlings. Biochemistry (Mosc).

[10] Pastore D., Trono D., Laus M.N., Di Fonzo N., Flagella Z. (2007). Possible plant mitochondria involvement in cell adaptation to drought stress. A case study: Durum wheat mitochondria. J. Exp. Bot..

[11] Gill S.S., Tuteja N. (2010). Reactive oxygen species and antioxidant machinery in abiotic stress tolerance in crop plants. Plant Physiol. Biochem..

[12] Beckman K.B., Ames B.N. (1998). The free radical theory of aging matures. Physiol. Rev..

[13] James A.M., Murphy M.P. (2002). How mitochondrial damage affects cell function. J. Biomed. Sci..

[14] Yun J., Finkel T. (2014). Mitohormesis. Cell Metab..

[15] Lázaro J.J., Jiménez A., Camejo D., Iglesias-Baena I., del Martí M.C., Lázaro-Payo A., Barranco-Medina S., Sevilla F. (2013). Dissecting the integrative antioxidant and redox systems in plant mitochondria. Effect of stress and S-nitrosylation. Front. Plant Sci..

[16] Liberatore K.L., Dukowic-Schulze S., Miller M.E., Chen C., Kianian S.F. (2016). The role of mitochondria in plant development and stress tolerance. Free Radic. Biol. Med..

[17] Møller I.M., Rasmusson A.G., van Aken O. (2021). Plant mitochondria—Past, present and future. Plant J..

[18] Porcher A., Kangasjärvi S. (2024). Plant biology: Unlocking mitochondrial stress signals. Curr. Biol..

[19] Mouritsen O.G. (2010). The liquid-ordered state comes of age. Biochim. Biophys. Acta (BBA)-Biomembr..

[20] Morigaki K., Tanimoto Y. (2018). Evolution and development of model membranes for physicochemical and functional studies of the membrane lateral heterogeneity. Biochim. Biophys. Acta (BBA)-Biomembr..

[21] Wang H.-Y., Bharti D., Levental I. (2020). Membrane heterogeneity beyond the plasma membrane. Front. Cell Dev. Biol..

[22] Kuimova M.K. (2012). Mapping viscosity in cells using molecular rotors. Phys. Chem. Chem. Phys..

[23] Simons K., van Meer G. (1988). Lipid sorting in epithelial cells. Biochemistry.

[24] Lagerholm B.C., Weinreb G.E., Jacobson K., Thompson N.L. (2005). Detecting microdomains in intact cell membranes. Annu. Rev. Phys. Chem..

[25] van Meer G., Voelker D.R., Feigenson G.W. (2008). Membrane lipids: Where they are and how they behave. Nat. Rev. Mol. Cell Biol..

[26] Sezgin E., Levental I., Mayor S., Eggeling C. (2017). The mystery of membrane organization: Composition, regulation and roles of lipid rafts. Nat. Rev. Mol. Cell Biol..

[27] Zheng Y.Z., Berg K.B., Foster L.J. (2009). Mitochondria do not contain lipid rafts, and lipid rafts do not contain mitochondrial proteins. J. Lipid Res..

[28] Garofalo T., Manganelli V., Grasso M., Mattei V., Ferri A., Misasi R., Sorice M. (2015). Role of mitochondrial raft-like microdomains in the regulation of cell apoptosis. Apoptosis.

[29] Rozentsvet O., Nesterkina I., Ozolina N., Nesterov V. (2019). Detergent-resistant microdomains (lipid rafts) in endomembranes of the wild halophytes. Funct. Plant Biol..

[30] Nurminsky V.N., Nesterov V.N., Rosentsvet O.A., Rakevich A.L., Bukin Y.S., Kapustina I.S., Ozolina N.V. (2021). Analysis of lipid order in raft structures of mitochondrial membranes of halophytes with the aid of fluorescence microscopy. Biochem. (Mosc.) Suppl. Ser. A Membr. Cell Biol..

[31] Neuburger M., Journet E.P., Bligny R., Carde J.-P., Douce R. (1982). Purification of plant mitochondria by isopycnic centrifugation in density gradients of Percoll. Arch. Biochem. Biophys..

[32] Grabelnykh O.I., Yakovenko K.V., Polyakova E.A., Korsukova A.V., Stepanov A.V., Fedotova O.A., Zabanova N.S., Lyubushkina I.V., Pobezhimova T.P., Borovskii G.B. (2022). Functioning of mitochondria in transgenic potato tubers with the *gox* gene for glucose oxidase from *Penicillium funiculosum* during different storage periods. Russ. J. Plant Physiol..

[33] Lowry O.H., Rosebrough N.J., Farr A.L., Randall R.J. (1951). Protein measurement with the Folin phenol reagent. J. Biol. Chem..

[34] Nikiforov N.G., Ryabova A., Kubekina M.V., Romanishkin I.D., Trofimov K.A., Chegodaev Y.S., Ivanova E., Orekhov A.N. (2021). Two subpopulations of human monocytes that differ in mitochondrial membrane potential. Biomedicines.

[35] Lyubushkina I.V., Fedyaeva A.V., Stepanov A.V., Grabelnych O.I. (2021). High temperatures induce ROS generation and damage to respiratory activity in *Saccharum officinarum* suspension cells. J. Sib. Fed. Univ. Biol..

[36] Bagatolli L.A. (2006). To see or not to see: Lateral organization of biological membranes and fluorescence microscopy. Biochim. Biophys. Acta.

[37] Watanabe N., Goto Y., Suga K., Nyholm T.K.M., Slotte J.P., Umakoshi H. (2019). Solvatochromic modeling of Laurdan for multiple polarity analysis of dihydrosphingomyelin bilayer. Biophys. J..

[38] Harris F.M., Best K.B., Bell J.D. (2002). Use of laurdan fluorescence intensity and polarization to distinguish between changes in membrane fluidity and phospholipid order. Biochim. Biophys. Acta.

[39] Wheeler G., Tyler K.M. (2011). Widefield microscopy for live imaging of lipid domains and membrane dynamics. Biochim. Biophys. Acta.

[40] Owen D.M., Rentero C., Magenau A., Abu-Siniyeh A., Gaus K. (2011). Quantitative imaging of membrane lipid order in cells and organisms. Nat. Protoc..

[41] Guaragnella N., Di Noia M.A., Primavera A. (2024). Mitochondrial (dys) function: A double edge sword in cell stress response. Front. Cell Death.

[42] Hill S., Sataranatarajan K., van Remmen H. (2018). Role of signaling molecules in mitochondrial stress response. Front. Genet..

[43] Ukolova I.V., Borovskii G.B. (2023). OXPHOS organization and activity in mitochondria of plants with different life strategies. Int. J. Mol. Sci..

[44] Gaus K., Gratton E., Kable E.P., Jones A.S., Gelissen I., Kritharides L., Jessup W. (2003). Visualizing lipid structure and raft domains in living cells with two-photon microscopy. Proc. Natl. Acad. Sci. USA.

[45] Gaus K., Zech T., Harder T. (2006). Visualizing membrane microdomains by Laurdan 2-photon microscopy. Mol. Membr. Biol..

[46] Weber P., Wagner M., Schneckenburger H. (2010). Fluorescence imaging of membrane dynamics in living cells. J. Biomed. Opt..

[47] Wenzel M., Vischer N.O.E., Strahl H., Hamoen L.W. (2018). Assessing membrane fluidity and visualizing fluid membrane domains in bacteria using fluorescent membrane dyes. Bio-protoc..

[48] Levitan I. (2021). Evaluating membrane structure by Laurdan imaging: Disruption of lipid packing by oxidized lipids. Curr. Top. Membr..

[49] Gunther G., Malacrida L., Jameson D.M., Gratton E., Sánchez S.A. (2021). LAURDAN since Weber: The quest for visualizing membrane heterogeneity. Acc. Chem. Res..

[50] Pokorna S., Ventura A.E., Santos T.C.B., Hof M., Prieto M., Futerman A.H., Silva L.C. (2022). Laurdan in live cell imaging: Effect of acquisition settings, cell culture conditions and data analysis on generalized polarization measurements. J. Photochem. Photobiol. B Biol..

[51] Kumari R., Sharma P., Chaturvedi V., Pati A.K. (2025). Organic fluorophores for studying lipid membrane structures and dynamics. Chem. Asian J..

[52] Orlikowska-Rzeznik H., Krok E., Chattopadhyay M., Lester A., Piatkowski L. (2023). Laurdan discerns lipid membrane hydration and cholesterol content. J. Phys. Chem. B.

[53] Boyd M.A.,  Kamat N.P. (2018). Visualizing tension and growth in model membranes using optical dyes. Biophys. J..

[54] Zapata-Mercado E., Azarova E.V., Hristova K. (2022). Effect of osmotic stress on live cell plasma membranes, probed via Laurdan general polarization measurements. Biophys. J..

[55] Singh G., George G., Raja S.O., Kandaswamy P., Kumar M., Thutupalli S., Laxman S., Gulyani A. (2023). A molecular rotor FLIM probe reveals dynamic coupling between mitochondrial inner membrane fluidity and cellular respiration. Proc. Natl. Acad. Sci. USA.

[56] Knippenberg S., De K., Aisenbrey C., Bechinger B., Osella S. (2024). Hydration- and temperature-dependent fluorescence spectra of Laurdan conformers in a DPPC membrane. Cells.

[57] Watanabe N., Suga K., Slotte J.P., Nyholm T.K.M., Umakoshi H. (2019). Lipid-surrounding water molecules probed by time-resolved emission spectra of Laurdan. Langmuir.

[58] Dinic J., Biverståhl H., Mäler L., Parmryd I. (2011). Laurdan and di-4-ANEPPDHQ do not respond to membrane-inserted peptides and are good probes for lipid packing. Biochim. Biophys. Acta.

